# Support System to Improve Reading Activity in Parkinson’s Disease and Essential Tremor Patients

**DOI:** 10.3390/s17051006

**Published:** 2017-05-03

**Authors:** Franklin Parrales Bravo, Alberto A. Del Barrio García, Mercedes Gallego de la Sacristana, Lydia López Manzanares, José Vivancos, José Luis Ayala Rodrigo

**Affiliations:** 1 Faculty of Computer Science, Complutense University of Madrid, Av. Séneca, 2, 28040 Madrid, Spain; abarriog@ucm.es (A.A.D.B.G.); jayala@ucm.es (J.L.A.R.); 2 Neurology Department, “La Princesa” University Hospital, Calle de Diego Leon, 62, 28006 Madrid, Spain; mariamercedes.gallegosacristana@salud.madrid.org (M.G.d.l.S.); lydia.lopez@salud.madrid.org (L.L.M.); jvivancos@neurogps.com.es (J.V.)

**Keywords:** Parkinson’s disease, essential tremor, e-health, user experience

## Abstract

The use of information and communication technologies (ICTs) to improve the quality of life of people with chronic and degenerative diseases is a topic receiving much attention nowadays. We can observe that new technologies have driven numerous scientific projects in e-Health, encompassing Smart and Mobile Health, in order to address all the matters related to data processing and health. Our work focuses on helping to improve the quality of life of people with Parkinson’s Disease (PD) and Essential Tremor (ET) by means of a low-cost platform that enables them to read books in an easy manner. Our system is composed of two robotic arms and a graphical interface developed for Android platforms. After several tests, our proposal has achieved a 96.5% accuracy for A4 80 gr non-glossy paper. Moreover, our system has outperformed the state-of-the-art platforms considering different types of paper and inclined surfaces. The feedback from ET and PD patients was collected at “La Princesa” University Hospital in Madrid and was used to study the user experience. Several features such as ease of use, speed, correct behavior or confidence were measured via patient feedback, and a high level of satisfaction was awarded to most of them. According to the patients, our system is a promising tool for facilitating the activity of reading.

## 1. Introduction

Parkinson’s Disease (PD) is a degenerative and progressive disorder with an unknown etiology, an overall incidence of 20 cases per 100,000 people and a prevalence of 150 per 100,000 people [1]. It represents the second most frequent neurodegenerative disease among the population after Alzheimer’s Disease, being associated with a significant increase in personal, social and labour disability [2]. This disease mainly causes the loss of dopaminergic neurons belonging to the substantia nigra and the consequent deficit when synthesizing dopamine. Dopamine is an essential neurotransmitter that regulates the basal ganglia function, which is involved in the management and coordination of movement [3]. Typically, PD causes three fundamental motor symptoms that affect both body trunk and limbs and around which its diagnosis is built: tremor, rigidity and bradykinesia. This last symptom is defined as the slowness of movement, with a decrease in amplitude too.

Essential Tremor (ET) is the most frequent movement disorder in adults. The population prevalence is estimated at around 0.4% [4], a percentage that increases exponentially for people older than 65. Specifically, it ranges between 4.6% and 14.3% for this population subset [5]. It has been considered a benign disease caused by the imbalance among different excitatory and inhibiting neurochemicals belonging to the central nervous system, but it can lead to considerable physical and psychosocial disability. It is characterized by the presence of oscillatory rhythmic movements while an antigravitational position is being held. In total, 90% of patients suffer these tremors in the upper limbs, although it can also affect other parts of the body such as the head, the lower limbs or even the voice [6].

Oscillatory rhythmic movements and bradykinesia are arguably the more disabling and disturbing consequences of PD and ET, as patients cannot usually perform conventional actions, such as reading a book, for instance [7]. In fact, patients have difficulties reading a physical book when the font size is small, and with trying to turn the pages. As a result of this disability while performing conventional easy tasks, patients become highly depressed [8,9], which makes tackling the disease more difficult.

The main problems while reading a book are derived from the aforementioned tremor, rigidity and bradykinesia. Due to axial rigidity, small fonts [10] are quite difficult to read. Moreover, patients with neurological problems cannot turn a single page with full precision. Usually, several pages are jointly turned. A possible solution to address the small font problem is to obtain books with larger fonts, or to use enlarged authorized photocopies to make the content more visible. Nevertheless, the turning of pages is a major problem that still must be solved. In this sense, ebooks could be a solution. Nonetheless, the electronic format requires zooming in on the page to be read, which represents a difficult action for people suffering from PD or ET. Netbooks and eReaders are other devices that allow reading books in digital format. These devices involve the use of technology and their adoption depends on how difficult is to learn a new technology [11]. Regarding the eReader limitations, it is necessary to mention that not all of them offer a good resolution for graphics, tables and diagrams. In this sense, the Netbook offers more attractive options for its connectivity, sharing applications and others. Nevertheless, some old books, study books and magazines are not available in a digital version [12]. Furthermore, none of the aforementioned approaches are able to properly tackle the shaky movements of the hands of ET and PD patients.

In addition to this, there are several scientific studies arguing that it is more difficult to understand text in digital format than in conventional books. Poorer comprehension, and slow reading are problems associated with ebooks. For example, Mangen et al. [13] concluded that the brain has to face additional difficulties which are not present when using a conventional book, such as learning to cope with a text with which we cannot physically interact. Moreover, other works [14,15,16] have shown that physical books provide a sense of control over the text and object manipulation (underlining, bending, etc.) that helps us better understand the text.

Hence, in this paper, we propose a low-cost platform to support reading for patients with tremor in their hands. This system provides an interface for patients to facilitate its use when selecting and turning pages. The features of the platform have been carefully studied, as shown in the experiments, improving upon the previous systems with a similar purpose. Moreover, this work has been evaluated on ET and PD patients in order to collect data about their satisfaction with the usability of the system.

The rest of the paper is organized as follows. The related work section reviews the state-of-the-art in the e-health field and its applications for improving the quality of life and detection of PD and other neurological diseases. The system description section shows the design of the device and its main functionalities. The experimental section describes the performance evaluations of the platform and the degree of satisfaction achieved by the system. Finally, we offer our final remarks and present our future lines of work in the conclusion section.

## 2. Related Work

E-health applications adopt two principal approaches: facilitating clinical decision making (involving experts in making clinical decisions) and improving the quality of life through different devices to support control or tackle difficulties of patients.

Several projects have been carried out in order to improve the quality of life, for example, a mobile speech therapy application is evaluated to provide visual feedback about voice volume in PD patients. In this application, an explicit signal for adjusting the patient’s voice volume to mitigate communication difficulties is emitted [17]. A robot-assisted gait therapy with the Lokomat system has been tested for increasing the gait speed, stride length and foot clearance during ground walking in a PD patient [18]. Moreover, robot-assisted arm training has been analyzed for improving upper limb function in patients with PD [19]. In addition, an upper-limb power-assisted robot with tremor suppression control in ET and PD patients is described in [20]. Other works present a similar approach for tremor suppression [21,22,23].

Regarding platforms for supporting reading, there is the Bookreader 2 approach [24]. It consists of two large servo motors whose purpose is to lift and turn the book’s pages. It provides an automated Optical Character Recognition (OCR) tool for physical books and it works with a camera that handles pages and reads them. However, this platform lacks some desirable features. For instance, a tactile interface needs to be implemented to handle the hand tremor. Another limitation is the low pressure level in the lifting arm. Consequently, this platform does not provide good control over the first and last pages of books. In addition, it does not consider the degree of inclination of the book to facilitate the visualization of pages for PD and ET patients because of the vertical structure for supporting the camera.

The development of this project has been based on a Lego EV3 platform [25]. This kit is composed of many Lego plastic blocks, several sensors and actuators, and a main block or brick which can be easily programmed [26]. The major advantage of Mindstorms is its capability to quickly build robotic projects, prototypes and complete systems by only joining a relatively small number of Lego building pieces. Lego components are very easy to assemble, as well as being very durable and economically affordable [27].

The decision to use the Lego EV3 kit instead of Raspberry Pi (Brick Pi) or Arduino is based on two reasons. First, Lego Mindstorms EV3 is a low-cost kit that can be easily adapted to the patient’s requirements. Hence, it is not necessary to pay a high cost for an ad-hoc robotic implementation. Secondly, as was previously mentioned, the major advantage of Mindstorms is its capability to quickly build robotic projects, prototypes and complete systems by only joining a fair amount of building Lego pieces. All sensors/actuators are integrated, while in the case of Arduino or Raspberry Pi, components need to be bought separately, calibrated, etc. On the other hand, however, a Lego EV3 kit is not a guaranty of perfect behavior; the design must still be done regardless of the platform.

Therefore, we propose a low-cost system that is able to interact with neurological patients through a tactile interface. Furthermore, our system works on inclined surfaces in order to facilitate the reading process for patients. In addition, we have studied different types of pages, location of the arms, battery duration, etc, thus providing a complete platform for dealing with PD and ET patient problems when reading a book.

## 3. System Description

**Figure 1:**
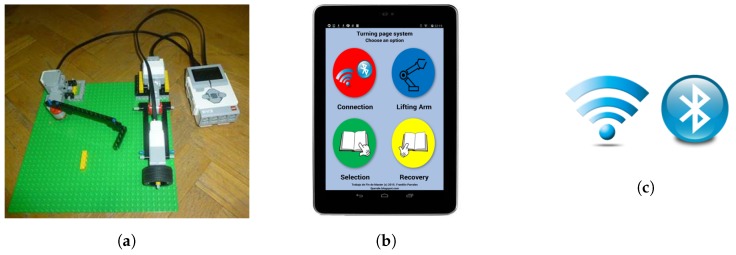
Components of this project. (**a**) Robotic arms; (**b**) Remote control system; (**c**) Bluetooth or Wi-Fi connectivity.

Our system is composed of the following:

- The hardware elements belonging to the Lego Mindstorms kit, as depicted in Figure 1a.
- A tactile interface developed for Android systems, which has been designed considering the motor limitations of PD and ET patients, as shown in Figure 1b.
- The type of connectivity that enables the remote control between the interface and the hardware components, as described in Figure 1c.

The protocol for the trial of the robotic arm is as follows. First, the assistant, who is the person that sets the configuration of the system, positions it properly with a suitable distance between the robotic arm and the book. The assistant is also responsible for establishing the Bluetooth or Wi-Fi connection between the Android device and the EV3 brick. Finally, this person will describe the operating instructions of the remote interface to the patient. Second, the patient handles the interface to turn the pages in order to read.

**Figure 2:**
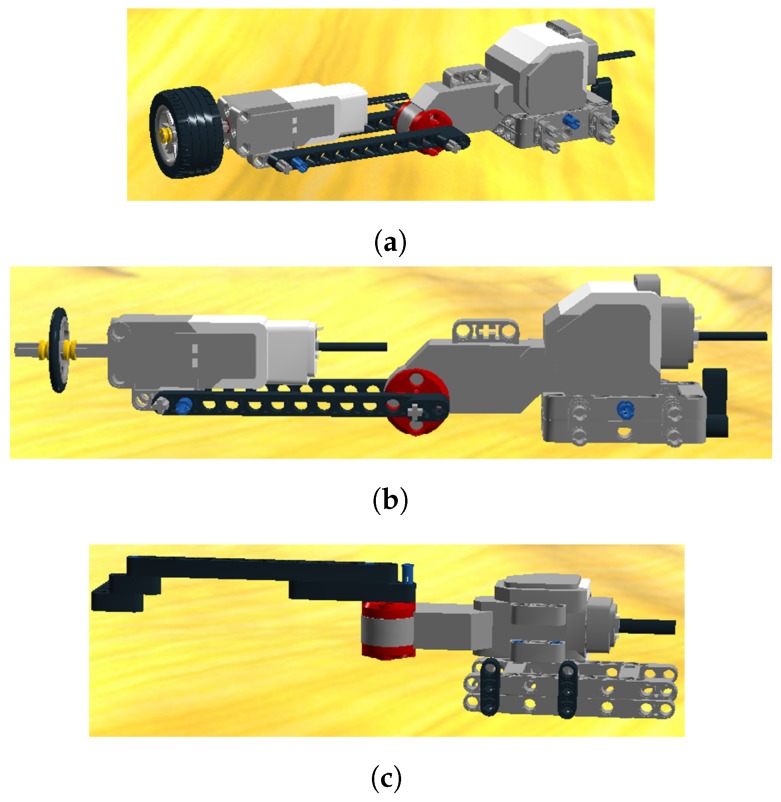
Lifting and turning arms. (**a**) Lifting arm with thick wheel; (**b**) Lifting arm with thin wheel; (**c**) Turning arm.

Two robotic arms have been built. The lifting arm has the function of handling a page. The turning arm is responsible for turning the page. In comparison with the Bookreader 2 project, the lifting arm includes a large servo motor for allowing both up and down movements. These movements will handle the pressure on the page, contributing to a finer page selection and to the recovery method in case of a bad page selection. Furthermore, the pressure handling allows a higher degree of precision when working in an inclined scenario. In our platform, we have also considered a thin wheel, as shown in Figure 2b, for selecting glossy and lightweight pages. This type of page presents less precision when using the conventional thick wheel described in Figure 2a. The turning arm consists of a large Lego studless beam joined to a large servo motor and other small pieces. Its design is depicted in Figure 2c.

Regarding the patient interaction with the platform, the hardware is not suitable for direct use by neurological patients because the brick buttons and screen are quite small, as in the case of eReaders. As a result, PD and ET patients find it difficult to perform an accurate pressure. In fact, the precision required to go to the following page is a big problem for them. For these patients, it is much easier to target a large colored button on the screen, rather than pressing a tiny button just one time. The rebound problems that occur due to their shaky hands when pressing the buttons are controlled by our developed software.

**Figure 3:**
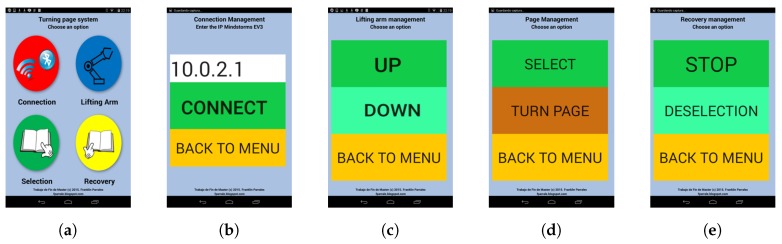
Screenshots of the user interface proposal. (**a**) Main menu; (**b**) Connection menu; (**c**) Lifting arm control; (**d**) Page selection options; (**e**) Recovery options.

The user interface depicted in Figure 3 is composed of five screens. It allows adaptation of the font size to cater for the patient’s motion and vision difficulties [10]. This involves the use of large buttons and fonts. Maximizing the use of the whole screen is important for making the interactions easier. Finally, the PD and ET patient’s visual difficulties can be minimized by using different colors for the button backgrounds.

The main menu, which is the first screen, is depicted in Figure 3a. It is composed of four buttons for connection, which lead to the lifting arm menu, the page selection menu and the page recovery menu. The connection screen has been designed for configuring the connection between the EV3 brick and the Android device. This must be done by the assistant. This screen is shown in Figure 3b. The third screen controls the lifting arm movements. As shown in Figure 3c, two actions can be selected in order to move the said arm up or down. The fourth screen controls the selection and turning of pages, and is shown in Figure 3d. The fifth screen controls the deselection of pages as well as the stopping of the selection wheel. This screen is shown in Figure 3e.

## 4. Experimental Section

Several tests have been performed with the reading support system for PD and ET patients. These experiments have consisted of counting the number of failures when the system turns a page; analyzing the battery use; and studying the system’s average response time in seconds. In addition, experiments on an inclined surface have been considered. Furthermore, a bookreader-based design has been used for comparison too. Finally, we have evaluated the system and collected data from twenty-six PD and ET patients at “La Princesa” Hospital in Madrid.

### 4.1. Error Rate

**Figure 4:**
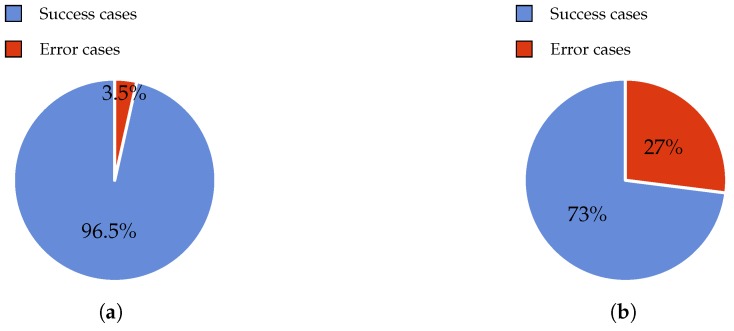
Error rate using non-glossy surface paper. (**a**) With thick wheel model; (**b**) With Bookreader 2 model.

In this experiment, 200 observations were conducted in order to obtain the error rate. This experiment was tested with 80 gr/m${}^{2}$ and A4 size paper. The A4 paper was selected because nowadays it is the most extended for commercial use. In fact, this format is very similar to the size of old book pages (big fonts and images) that elderly people usually read. The results are shown in Figure 4a. When using the proposed design, i.e., thick wheels, we achieved 193 successful cases in contrast to seven failures. The error rate was thus 3.5%. Five of these failures were solved by deselecting and selecting the page once again. The remaining two errors were solved by moving the roller down to apply more pressure.

In the same scenario, the Bookreader 2 model obtains worse results. These can be observed in Figure 4b. Specifically, 146 successful cases and 54 failures were obtained. It is very important to mention that this model cannot correctly select the first and the last pages (four cases) of the book because it does not have a servo motor for adding pressure as our thick wheel model does. Regarding the rest of the errors, the majority of failure cases (45 cases) consisted of two pages being jointly selected. Hence, this justifies the use of the second motor in our design. The rest of the failures (five cases) consisted of not lifting the page properly, prior to turning it. Therefore, the introduction of a recovery mechanism is necessary, as highlighted by these results.

**Figure 5:**
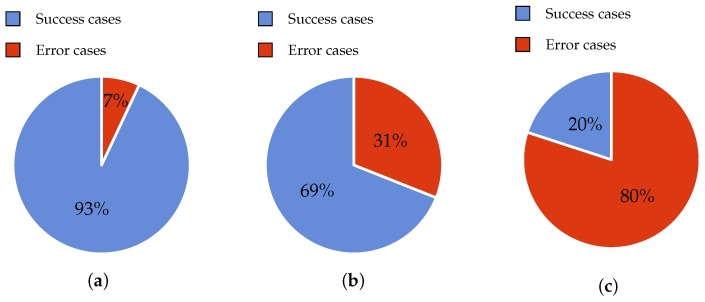
Error rate using a glossy surface paper. (**a**) With the thin wheel model; (**b**) With the thick wheel model; (**c**) Using bible-like paper and the thin wheel model.

The thin wheel model does not work with this type of paper. In general, this model does not work unless glossy and lightweight pages are used. In order to check the suitability of our approach, a magazine with paper of A4 size and 65 gr/m${}^{2}$ and a glossy surface was tested. The results of these tests can be observed in Figure 5a,b. In this case, the use of the thin wheel model is more effective than the thick wheel one because it produces less failures (7% vs. 31%) when selecting the pages.

Paper possessing a weight below 65 gr/m${}^{2}$ is not recommended because of multiple page selection failures. For instance, the Bible (50 gr/m${}^{2}$) was tested using the thin wheel. As can be observed in Figure 5c, the error rate is high (80%) with this type of paper. With the use of the thick wheel or the Bookreader 2 model, the number of errors is close to 100%.

### 4.2. Battery Usage

The main objective of this experiment is to establish the number of pages that can be correctly turned before recharging the battery. We should mention that the EV3 battery voltage level ranges from 8.0 to 6.5, where 8.0 indicates that the battery level is high while 6.5 is the lowest battery level prior to turning off the EV3 brick. Hence, at this level, the battery needs to be recharged.

**Figure 6:**
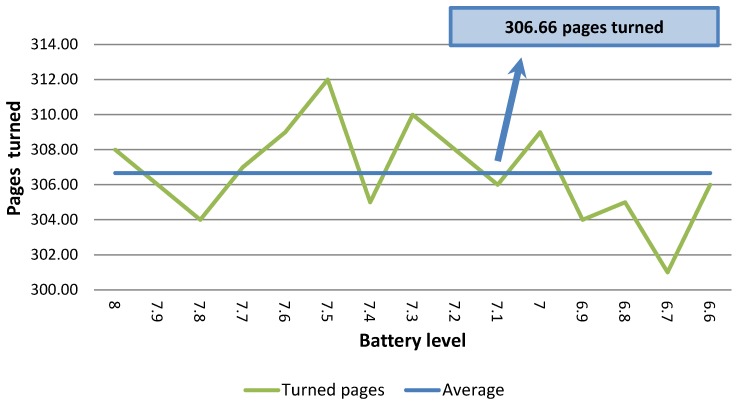
With the thick wheel model and non glossy 80 gr/m${}^{2}$ pages.

The thick wheel model was tested using 80 gr/m${}^{2}$, A4 size paper without a glossy surface. The results are depicted in Figure 6. The average number of pages turned was close to 307. Overall, it was possible to turn around 4600 pages before recharging the battery.

**Figure 7:**
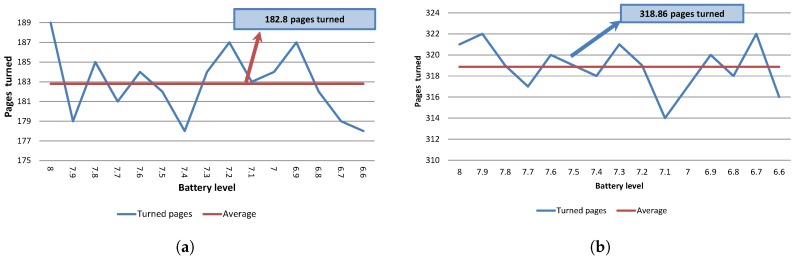
Battery consumption for 65 gr/m${}^{2}$ pages. (**a**) Battery usage with the thin wheel model and glossy pages; (**b**) Battery usage with the thick wheel model and non-glossy pages.

The thin wheel model was evaluated too, but considering glossy A4 pages with 65 gr/m${}^{2}$ weight, as depicted in Figure 7. With this model, on average it is possible to turn around 183 pages per battery consumption level. Overall, it was possible to turn 2742 pages before recharging the battery. The results are shown in Figure 7a. The main reason for this decrease stems from the fact that the thin wheel requires a longer time to select a page. In the case of non-glossy A4 pages, the thin model does not work properly. When using the thick model in this scenario but with non-glossy paper, on average around 319 pages per battery consumption level are obtained. The results are depicted in Figure 7b. As can be observed, there is a slight increase in terms of pages turned with respect to 80 gr/m${}^{2}$ pages, which is to be expected because of the weight difference. In conclusion, the thin wheel model is necessary for dealing with glossy surfaces, while the thick wheel model is better for non-glossy pages.

### 4.3. Average Time When Turning a Page

The goal of this experiment is to evaluate the time required by the platform users to turn the pages. A very important point to consider is that the participants in these experiments are users without motor limitations. Thus, in the case of PD patients, the time could be greater depending on the severity of the disease.

**Figure 8:**
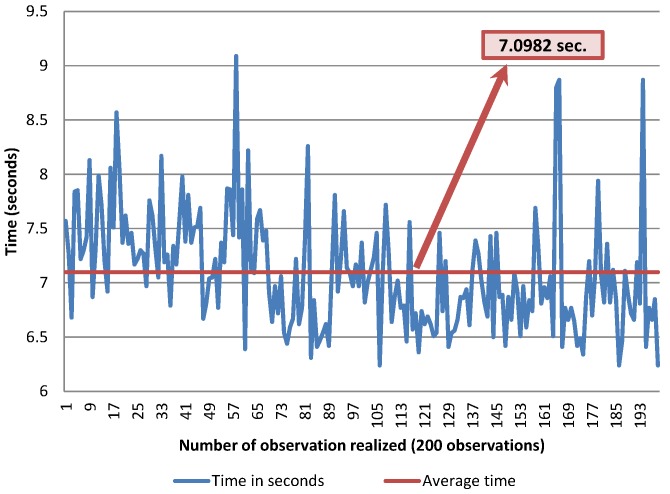
Average time with the thick wheel model.

The thick wheel model was tested with 200 observations (only successful turning cases), using 80 gr/m${}^{2}$ and A4 sized pages. The time was measured in seconds, using a chronometer. The overall elapsed time is measured from the page selection action to the moment when the lifting arm has completed its downwards movement after turning a page. The average time obtained is 7.1 s per page, which means that the system is able to turn around 507 pages per hour. These results are shown in Figure 8.

**Figure 9:**
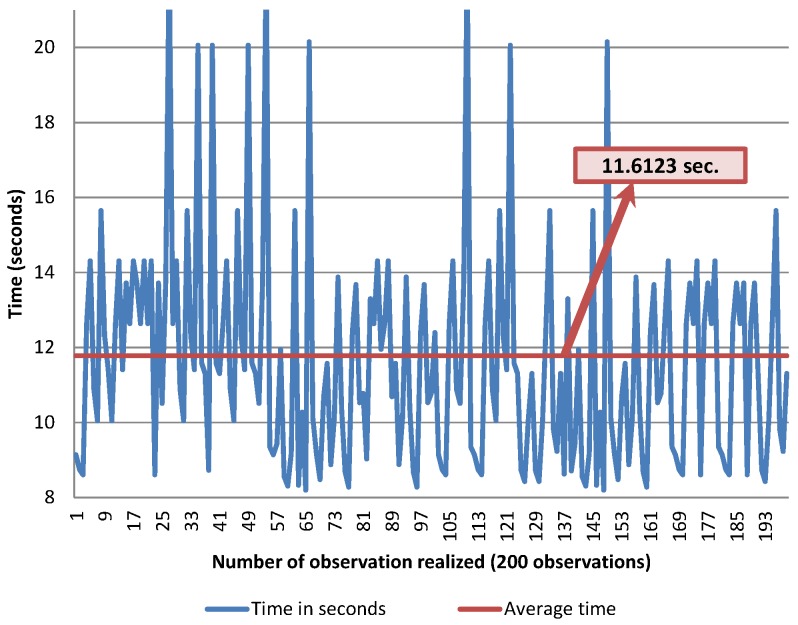
Average time with the thin wheel model.

Different results were obtained in the case of the thin wheel model with 65 gr/m${}^{2}$ and A4 sized glossy paper. The average time is 11.6 s per page, which is higher than with the thick wheel, meaning that the system is able to turn nearly 310 pages per hour. The results are shown in Figure 9. The increase in time is a consequence of the page selection time augmentation when using the thin wheel model. Nevertheless, it must be remembered that the thin wheel model achieves greater precision when turning glossy lightweight pages.

**Figure 10:**
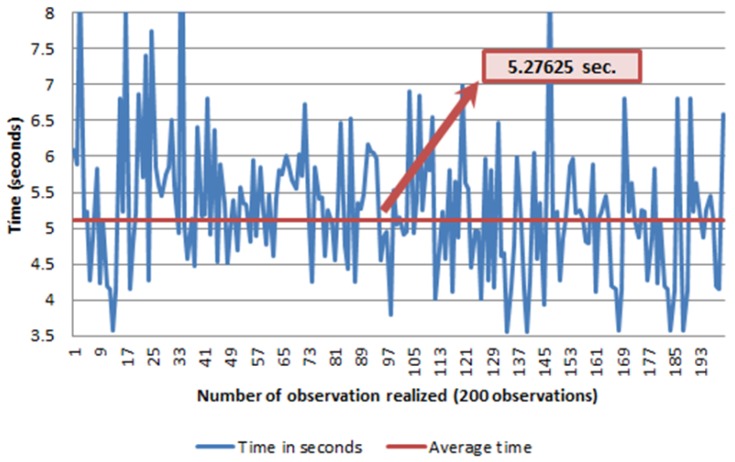
Average time with the Bookreader 2 model.

The Bookreader 2 model was tested too, using 80 gr/m${}^{2}$, A4 sized paper without a glossy surface. The results are shown in Figure 10. The average time is less than that of the other models, as the selection action does not involve two servo motors, but just one. However, it should be noted that the error rate is higher than that of our proposal. Furthermore, this noticeable average time decrease is due to the fact that only correctly turned pages are being considered in this study.

### 4.4. Arms Location

**Figure 11:**
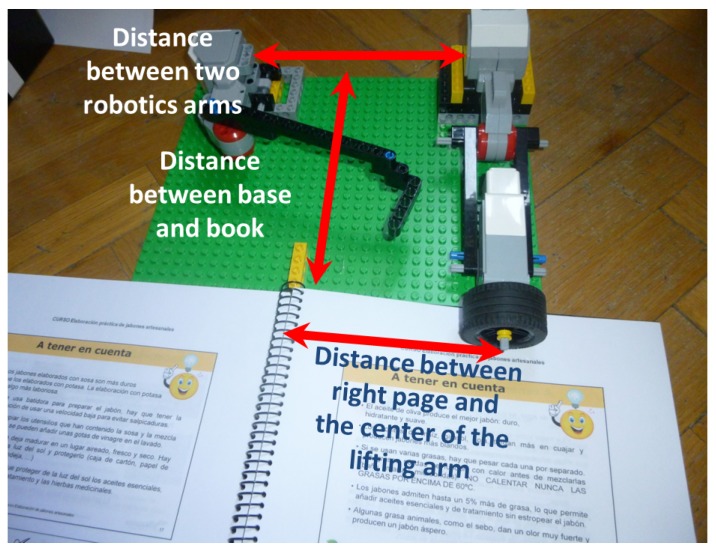
Location of distances between book and robotic arms.

It is very important to establish a relationship between the book dimensions and the position of the robotic arms in order to ensure successful page selection. This test was carried out on different ring bound books because of the stability that they offer when a page has to be turned to the left. Each test was performed on 100 A4 sized pages (21 × 29.7 cm). In addition, glossy and non-glossy surfaces were used, employing the thin wheel model for glossy pages and the thick wheel model for non-glossy pages. Figure 11 shows how the system was deployed and the three distances that were evaluated. The results are shown in Table 1.

The distance between the robotic arms is set to 15 cm, and the distance between the book and the Lego base is set to 19 cm. The reason for using these distances is the fact that, with other values, the book is damaged when turning pages or the wheel position obstructs the view of the pages. With a larger distance, we have observed that it is not possible to correctly perform page selection with A4 sized pages.

According to the results obtained, it can be concluded that the most suitable distance between the paper and lifting arm for A4 non-glossy paper is 12 cm, as this achieves the best results in terms of average time and success rate for pages of 65, 70 and 80 gr/m${}^{2}$ in weight. In the case of lightweight and glossy paper, the tests were performed with 65, 70 and 80 gr/m${}^{2}$ A4 pages, and the most suitable distance in this case is 10 cm because this leads to the best results in terms of success rate.

With the same distances between the robotic arms (15 cm) and between the book and the Lego base (19 cm), other unusual scenarios were evaluated. Specifically, the following results in terms of lifting arm distance were obtained:

- A distance of 14 cm between the lifting arm and the right page in the case of 20.5 × 14 cm non-glossy pages with a weight of 120 gr/m${}^{2}$.
- A distance of 12 cm for a width of 16.7 cm and a weight of 80 gr/m${}^{2}$ in non-glossy pages.

In addition, the optimum arm position depends on the paper dimensions and the gloss, rather than the weight. In the most common case, i.e., A4, it seems that two optimum distances exist, namely 12 cm and 10 cm depending on the gloss, but in the general case there is not a straightforward relationship among these three parameters.

### 4.5. Testing on an Inclined Surface

**Figure 12:**
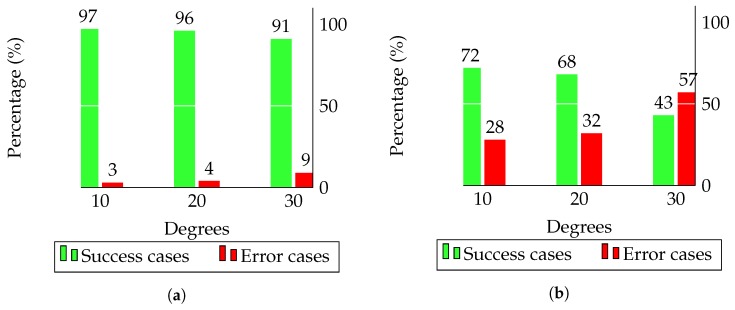
Error rate on a 10 , 20 and 30 degree inclined surface. (**a**) Thick wheel model; (**b**) Bookreader 2 model.

The goal of this experiment is to measure the error rate when the Bookreader 2 model and the proposed model are turning pages in a scenario with a certain slope. These two models were evaluated with 10, 20 and 30 degrees of inclination, 100 non-glossy pages of A4 size and a weight of 80 gr/m${}^{2}$. The results are shown in Figure 12.

When the slope is moderate, i.e., 10 degrees, both approaches have similar results to those obtained on a non-inclined surface. However, the Bookreader 2 model produces significantly more failures with 20 degrees than when working on a horizontal surface (32% vs. 27%). In contrast to this accuracy loss, the proposed model maintains a similar error rate to that when working on a conventional non-inclined surface. Consequently, an inclined scenario requires the system to add a certain pressure to the pages, which motivates the introduction of the second motor in our lifting arm, in contrast to the Bookreader 2 design.

### 4.6. User Satisfaction

The purpose of this experiment is to study the user experience when employing the proposed system. In order to obtain real feedback, the system was used by several patients that are being treated by the Neurology Department at “La Princesa” University Hospital in Madrid. The protocol of the study was approved by the hospital’s Research Ethic Committee. (The hospital’s Research Ethic Committee decided that no ethical aspects needed to be approved as the study was non-intrusive.) The patients signed their corresponding informed consent forms. It must be noted that the proposed platform could not be compared with other platforms such as the eReaders because the elderly people that took part in the experiment did not feel comfortable with them. In fact, they were asked to try an eReader during the tests, but they were very reluctant to utilize the device.

Our sample was composed of 26 patients, of whom 14 suffered from PD (54%) and 12 from ET (46%), with their upper limbs affected by these diseases. Individuals with functional repercussion of their disease demonstrated by the Schwab and England (SE) scale [28] for PD and the Fahn–Tolosa–Marín (FTM) scale for ET [29] were selected.

In PD, an 80% SE score implies that the patients have a degree of disablement that doubles the time required to perform the basic activities of daily living, while a score lower than 80% means that the patient is not fully independent. In ET, the FTM metric comprises three parts that rank as localization/severity of the tremor (part A), specific motor tasks (part B), and functional disability as a consequence of the tremor (part C). Hence, thanks to part C of the FTM scale, it is possible to grade this functional disability for performing daily tasks, such as eating, writing, washing, etc. Although there does not exist any definition of cut-off points regarding this scale, only those ET patients possessing an FTM score ≥4 were selected. According to the medical criteria, FTM values ≥4 imply that the quality of life of the patient is significantly affected.

Those individuals presenting either a visually, psychiatrically or cognitively disabling pathology were discarded. The level of cognitive impairment was measured through the mini-mental scale examination (a widespread technique in this scenario).

Five items were measured from 1 to 5 using a Likert scale [30] with the purpose of understanding the users’ opinions regarding interaction with the system. In this case, the thick wheel model was employed. Numbers from 1 to 5 represent the following values, respectively: very disappointed, disappointed, acceptable, satisfactory, excellent. A specific feature of the system is considered to be approved when the score obtained is greater than, or equal to, 3. The system features that were evaluated are ease of use (A); speed (B); correct behaviour of the system (C); confidence to use it again (D); and interface suitability (E). The survey can be found in Appendix A.

In addition, other objective data were collected from the patients: how long since the disease was detected in years (DD, Disease Detected); the age of the patient in years; the time in seconds to turn ten pages when using their hands (TT, Turning Ttime) and when using the proposed system (TTS, Turning Time with System). These metrics allow us to know the patient’s degree of difficulty when turning pages, as well as the potential benefits when employing our system. Moreover, another column specifies the disease of the patient: PD for Parkinson Disease and ET for Essential Tremor. It must be noted that the SE and FTM (only part C) values are provided for PD and ET patients, respectively, utilizing NA (Not-Applicable) where applicable. The data collected are presented in Table 2.

According to the this, the average time since detection of the disease and the average age of our population are 9.2 and 73 years, respectively. In terms of time, when the pages are manually turned, there is an average delay of 54 s, and around 75 s when the system is utilized. Therefore, this system provides a time gain for patients whose manual turning page time is greater than 7.5 s. This time can, however, be reduced at the expense of losing accuracy and the impossibility of recovering from failures, as in the Bookreader 2 project [24]. Another possibility consists of accelerating the movement of the turning arm. Nevertheless, by doing this, we have observed that pages are prone to being spoilt or torn.

Overall, three patients clearly benefited from the system’s employment (patients 5, 25 and 26), with time-saving benefits when turning the pages of a book. Two of these patients suffered from PD and the other from ET. Moreover, there are six more patients with a |TTS−TT|≤8s (patients 1, 2, 9, 10, 11 and 19). As the patients are old and in general unfamiliar with technology, we strongly believe that after some days practising with the system, this subset would easily obtain a greater benefit.

In the ET patients group, we observed that the patients who benefit the most from the system are those possessing the highest FTM scores.

Analogously, the most affected PD patients should intuitively be the ones to benefit most from the system. However, five patients presenting just a certain functional dependency (SE score <80%) belong to the aforementioned subset of those that benefit or are likely to benefit with more training (patients 9, 11, 19, 25 and 26). From a clinical point of view, it is well known that the highest degree of functional disability is associated with axial symptoms, such as slowness or rigidity when moving the body trunk and the main joints, which makes it difficult to walk or perform positional turnings. On the other hand, the appendicular motor symptoms or those present in the limbs may also interfere with the quality of life of the patients, but typically to a lower degree. For these reasons, not all the PD patients find it difficult to turn the pages of a book and, therefore, several of them cannot improve their speed when performing this activity.

As has been mentioned above, the user satisfaction was measured via questions A to E, obtaining the following average values, respectively: 4.3, 3.9, 4.2, 4.1 and 4.5. If we split the population depending on the disease, the following values are obtained for PD patients: 4.4, 3.8, 4.4, 4.5 and 4.6; while for ET patients, the values are: 4.3, 3.9, 4.1, 3.7 and 4.4. In general, satisfaction is quite similar but for the confidence to use the system again (question D), the PD patients present a significantly higher acceptance than the ET ones (4.5 vs. 3.7).

**Figure 13:**
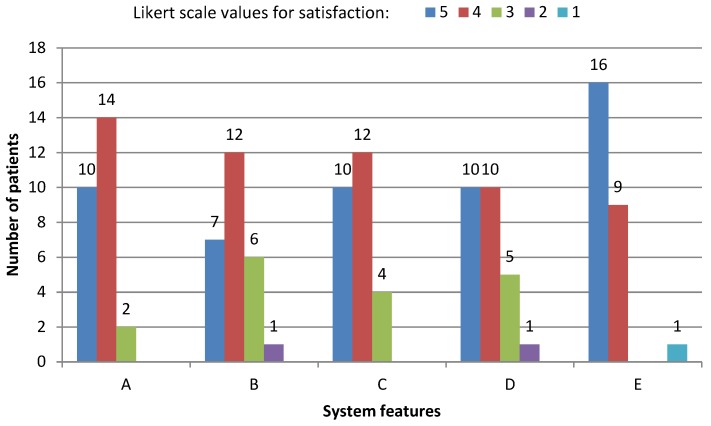
User satisfaction.

In Figure 13, the user satisfaction breakdown can be observed. According to both Table 2 and Figure 13, the degree of satisfaction ranges from 4 to 5 for the majority of the patients. Only three system features were given less than 3 points by the same patient. This patient presents a low level of ET, as his associated part C FTM value is 5. In fact, he just needed 32 s to turn the 10 pages. Consequently, the low rating given to the system features can be understood as an outlier. Thus, in general, it is possible to establish a correlation between the time to turn the pages and user satisfaction, obtaining a higher level of satisfaction as the time to turn the 10 pages increases, especially concerning the system’s speed (question B).

Finally, it should be noted that the system feature with the highest score is the interface. The patients mentioned that the large size of the buttons, the color difference between the options and the interaction with the tablet are very interesting features that improve the usability of the system.

## 5. Conclusions

A low-cost system for helping patients to read books has been proposed. This system was built using the Lego Mindstorm EV3 kit and an Android-based tablet. The proposed system works with two robotic arms, one for selection and another for turning pages. The addition of a second servo motor allows a higher degree of precision in comparison with the state-of-the-art solutions. As the experiments show, our system guarantees a good performance for different types of paper as well as inclined surfaces.

The proposed model has obtained positive usability results after testing it with patients under treatment at the Neurology Department of “La Princesa” Hospital in Madrid, which encourages us to continue working in this direction. The implementation of a voice command interface as well as the reduction in size of the system are future lines of work to consider. Furthermore, more tests are being contemplated for supporting different paper dimensions.

## Figures and Tables

**Table 1:** Average time and success rate for different weight/gloss/distance configurations.

Weight (gr/m^2^)	Glossy Page	Distance between the Center of the Lifting Arm and Right Page (cm)	Average Time	Success Rate
80	Yes	10	12.8562	94%
80	Yes	11	12.9456	83%
80	Yes	12	13.0127	75%
80	Yes	13	12.8754	69%
80	Yes	14	12.8652	57%
80	No	10	7.0124	87%
80	No	11	7.0235	92%
80	No	12	7.0982	96%
80	No	13	7.1623	92%
80	No	13	7.2156	91%
70	Yes	10	11.2156	92%
70	Yes	11	11.6879	82%
70	Yes	12	11.9564	74%
70	Yes	13	11.8754	65%
70	Yes	14	11.6523	48%
70	No	10	7.2156	93%
70	No	11	7.3956	93%
70	No	12	7.4181	95%
70	No	13	7.4536	91%
70	No	14	7.5689	90%
65	Yes	10	11.4948	93%
65	Yes	11	11.2244	84%
65	Yes	12	11.8509	83%
65	Yes	13	11.2208	62%
65	Yes	14	11.1031	54%
65	No	10	8.1256	95%
65	No	11	8.2378	96%
65	No	12	8.2965	97%
65	No	13	8.3546	92%
65	No	14	8.5645	88%

**Table 2:** Patients’ feedback.

Patient	Age	DD	Disease	SE	FTM	TT	TTS	A	B	C	D	E
1	77	20	ET	NA	7	70	71	5	3	4	4	4
2	86	10	ET	NA	15	90	82	5	5	4	5	5
3	75	1	PD	80	NA	60	79	5	4	4	4	4
4	83	4	PD	80	NA	48	74	5	4	5	5	5
5	82	7	ET	NA	17	146	81	4	5	5	3	5
6	78	7	ET	NA	8	27	72	4	3	3	3	4
7	64	4	ET	NA	5	32	75	4	2	3	2	1
8	84	2	PD	70	NA	44	73	5	4	4	5	5
9	82	3	PD	60	NA	65	71	5	4	3	4	5
10	81	4	ET	NA	12	80	74	5	4	4	3	4
11	70	4	PD	60	NA	67	71	4	5	4	4	4
12	73	9	ET	NA	6	32	75	4	4	5	4	5
13	64	10	PD	80	NA	17	72	4	3	5	4	5
14	71	35	ET	NA	7	36	79	4	3	4	3	5
15	56	27	ET	NA	11	43	81	3	5	5	5	5
16	51	10	ET	NA	4	12	73	4	5	4	3	5
17	61	10	PD	70	NA	57	83	4	4	5	5	5
18	67	7	ET	NA	6	23	76	5	5	5	5	5
19	85	4	PD	50	NA	74	72	4	4	4	5	4
20	73	12	PD	80	NA	29	74	3	3	4	4	4
21	91	4	PD	60	NA	19	72	4	3	4	4	4
22	62	5	PD	80	NA	45	74	4	5	4	5	5
23	60	20	ET	NA	8	22	73	4	3	3	4	5
24	71	8	PD	80	NA	17	77	5	3	5	5	4
25	72	4	PD	70	NA	124	75	4	4	5	4	5
26	81	9	PD	70	NA	140	75	5	3	5	5	5
Averages	73.1	9.2		70.7	8.8	54.6	75.2	4.3	3.9	4.2	4.1	4.5

## References

[1] De Lau L.M., Breteler M.M. (2006). Epidemiology of Parkinson’s disease. Lancet Neurol..

[2] De Rijk M.C., Launer L.J., Berger K., Breteler M.M., Dartigues J.F., Baldereschi M., Fratiglioni L., Lobo A., Martinez-Lage J., Trenkwalder C. (2000). Prevalence of Parkinson’s disease in Europe: A collaborative study of population-based cohorts. Neurologic Diseases in the Elderly Research Group. Neurology.

[3] Hornykiewicz O. (1998). Biochemical aspects of Parkinson’s disease. Neurology.

[4] Benito-León J., Louis E.D. (2007). Clinical update: Diagnosis and treatment of essential tremor. Lancet.

[5] Benito-León J., Louis E.D. (2011). Update on essential tremor. Min. Med..

[6] Benito-León J. (2011). Essential tremor: One of the most common neurodegenerative diseases?. Neuroepidemiology.

[7] Casamitjana C.F., García S., Méndez A.Z., Salazar M.H., Suárez S.S., Dávalos E.M., Ortiz C.G., Venegas J.B., Granados F.J., Cervantes J.H. (2007). Calidad de vida en pacientes con enfermedad de Parkinson y estimulación cerebral profunda. Med. Interna Méx..

[8] Mayeux R., Stern Y., Rosen J., Leventhal J. (1981). Depression, intellectual impairment, and Parkinson disease. Neurology.

[9] Cummings J.L. (1992). Depression and Parkinson’s disease: A review. Am. J. Psychiatry.

[10] Davidsdottir S., Cronin-Golomb A., Lee A. (2005). Visual and spatial symptoms in Parkinson’s disease. Vis. Res..

[11] Barnard Y., Bradley M.D., Hodgson F., Lloyd A.D. (2013). Learning to use new technologies by older adults: Perceived difficulties, experimentation behaviour and usability. Comput. Hum. Behav..

[12] Algoet C., Lerinckx D., Vandooren F. (2011). Using Mobile Devices for Reading eBooks: An Experiment with eReaders and Netbooks.

[13] Mangen A., Walgermo B.R., Brønnick K. (2013). Reading linear texts on paper versus computer screen: Effects on reading comprehension. Int. J. Educ. Res..

[14] Jabr F. (2013). Why the brain prefers paper. Sci. Am..

[15] Hillesund T. (2010). Digital reading spaces: How expert readers handle books, the Web and electronic paper. First Monday.

[16] Billingsley M.K. (2009). Proust and the Squid: The Story and Science of the Reading Brain. J. Am. Acad. Child Adolesc. Psychiatry.

[17] Nolan P.M. Development of a Mobile Speech Therapy Application-Encouraging Louder Communication in Parkinson’s Patients. Proceedings of the Medicine 2.0 Conference.

[18] Ustinova K., Chernikova L., Bilimenko A., Telenkov A., Epstein N. (2011). Effect of robotic locomotor training in an individual with Parkinson’s disease: A case report. Disabil. Rehabil. Assist. Technol..

[19] Picelli A., Tamburin S., Passuello M., Waldner A., Smania N. (2014). Robot-assisted arm training in patients with Parkinson’s disease: A pilot study. J. Neuro Eng. Rehabil..

[20] Kiguchi K., Hayashi Y., Asami T. An upper-limb power-assist robot with tremor suppression control. Proceedings of the 2011 IEEE International Conference on Rehabilitation Robotics (ICORR).

[21] Gallego J.Á., Ibanez J., Dideriksen J.L., Serrano J.I., del Castillo M.D., Farina D., Rocon E. (2011). A multimodal human-robot interface to drive a neuroprosthesis for tremor management. Trans. Syst. Man Cybern. Part C Appl. Rev..

[22] Riviere C.N., Thakor N.V. (1996). Modeling and canceling tremor in human-machine interfaces. IEEE Eng. Med. Biol. Mag..

[23] Pons J.L., Rocon E., Ruiz A.F., Moreno J.C. (2007). Upper-Limb Robotic Rehabilitation Exoskeleton Tremor Suppression.

[24] Dexter Industries (2014). BrickPi Bookreader 2. http://www.dexterindustries.com/BrickPi/projects/brickpi-bookreader-2/.

[25] Lego (2013). LEGO MINDSTORMS EV3. http://www.lego.com/en-us/mindstorms/about-ev3.

[26] Laamanen M. (2015). Architecture for Theatre Robotics. Master’s Thesis.

[27] Reshko G., Mason M.T., Nourbakhsh I.R. (2002). Rapid Prototyping of Small Robots.

[28] Schwab R.S., England A.C. (1969). Projection technique for evaluating surgery in Parkinson’s disease. Third Symposium on Parkinson’s Disease.

[29] Fahn S., Tolosa E., Marín C. (1993). Clinical rating scale for tremor. Parkinson’s Disease and Movement Disorders.

[30] Likert R. (1932). A technique for the measurement of attitudes. Arch. Psychol..

